# Analyzing Migration Restriction Regimes

**DOI:** 10.3389/fsoc.2021.610432

**Published:** 2021-04-13

**Authors:** Guillermina Jasso

**Affiliations:** Department of Sociology, New York University, New York, NY, United States

**Keywords:** international migration, U.S. immigration law, personal restriction, numerical restriction, consequences of migration restriction, visa backlogs and unauthorized migration, periodization of U.S. immigration history, creative immigration policy devices

## Abstract

This paper develops a framework for analyzing migration restriction regimes, and illustrates it with the case of U.S. immigration law and policy. Nation-states regulate the entry of foreign-born persons, and this regulation comprises three elements: the type of restriction, the apparatus of restriction, and the consequences of restriction. Restriction may be based on personal characteristics, numerical ceilings, or both. Personal restriction notices the characteristics of persons, using them as criteria for granting or denying admission. Numerical restriction places numerical ceilings on admissions. The apparatus of restriction may stipulate specific ceilings, whether some groups are exempt from the ceiling and, if so, by what criteria, and whether admission under the ceiling is first-come/first-served or by lottery or instead preferential and, if so, by what criteria. Two unintended consequences follow immediately: unauthorized migration (under both personal and numerical restriction); and visa-number backlogs (under numerical restriction). These in turn generate a range of policy devices: border enforcement, procedures for legalization and deportation, and procedures for clearing backlogs. Indeed, the history of a country's immigration law may be understood as a sequence of measures for first setting up the apparatus of restriction and then altering it in order not only to re-examine provisions of the initial setup but also to address unauthorized migration and visa-number backlogs. Viewing migration through this lens enables assessment of particular legislation and, more broadly, dynamics of a migration restriction regime, subject to world circumstances, including its possible inherent instability. The migration restriction lens also generates new metrics for a country's attractiveness and its innovativeness and creativity. To illustrate, the paper examines the migration restriction regime in the United States since the country's founding. Finally, the paper provides a checklist for a migration restriction setup that doubles as the basis for table shells for summarizing a country's migration restriction regime and its history.

## Introduction

Restriction is central to the history of international migration. Indeed, restriction is central to the human experience, playing out in a variety of social domains: whom to admit – to college, to particular employment, to an apartment building, to a neighborhood, to an honor society, to a club. Religions have rules of admission, elaborate rules for deciding, for example, who can become a Catholic, a Jew, a Muslim, etc. For countries, the stakes are high. As the American legislator Representative Peter W. Rodino put it, “Immigration and refugee policies… both reveal and define what kind of a nation we are and what kind of a nation we will become” (U.S. Select Commission on Immigration Refugee Policy, 1980a, p. 3).

Migration restriction operates in a variety of contexts, both within and across countries (i.e., both in internal migration and in international migration) – whom to admit as tourists, whom to admit for temporary sojourns, whom to admit for permanent settlement, and (in the case of international migration) whom to admit to citizenship and nationality. The framework introduced in this paper covers all migration contexts (and indeed is generalizable beyond migration). However, for concreteness, description is in terms of permanent immigration from one country to another. The section on Migration Restriction Regimes provides a brief overview of the framework for studying migration restriction regimes, describing three main elements – types of restriction, apparatus of restriction, and consequences of restriction. The section on Migration Restriction Regimes in the Broader Social Science Context considers the broader social science context, discussing matters of measurement and theory. Next, the section on Migration Restriction Regimes in the United States illustrates with the case of U.S. immigration law and policy. The paper concludes with a brief Afterword on possible creative policy devices and one dimension of personal restriction in the United States.

## Migration Restriction Regimes

### Two Types of Migration Restriction

There are two ways to restrict immigration – personal and numerical. A country may use personal criteria to screen prospective immigrants (for example, barring persons who are poor or illiterate) or numerical criteria (for example, setting an overall ceiling), leading to four possible restriction regimes. A priori, all four regimes are possible. One can envision a society with numerical restriction but no personal restriction (i.e., everyone is eligible but only a subset is admitted) or the opposite (i.e., only a subset is eligible and everyone in the subset is admitted). The two other regimes include the fully restricted regime with both personal and numerical restriction and the fully unrestricted regime. Thus, the two types of restriction lead naturally to four migration restriction regimes and thence, as will be seen below, to a periodization of the history of a country's immigration law.

### The Apparatus of Migration Restriction

Restriction is not easy. Restriction requires fitting together a set of moving parts. Consider the main parts of the apparatus, separately for personal and numerical restriction.

#### Elements of Personal Restriction

The main challenge is to define the set of personal characteristics that will be used to render prospective immigrants eligible or ineligible for legal permanent residence. If the characteristic is qualitative, like gender, eye color, nativity, religion, or native language, the decision must be made about which category or categories to favor or to bar. Should the destination country prohibit the immigration of blue-eyed persons? Or persons with certain illnesses? Or persons born in certain countries? Conversely, should the country accept only brown-eyed persons? Or persons with specified health characteristics? Or persons born in certain countries? Moreover, if origin country is to play a part, how should it be defined? As country of birth, or country of current or last residence, or country of citizenship? If the characteristic is quantitative, like wealth or age, the decision must be made which end of the continuum to bar and where to draw the line. Should the country prohibit the immigration of rich people, and where should it draw the line between rich and poor? Should the country prohibit the immigration of older persons, and where should it draw the line between young and old?

None of these questions is easy. And one can imagine that legislative bodies, as well as the citizenry, will have a diversity of views and spirited discussions. The documents of every policymaking body that has considered these questions display the great difficulties.

#### Elements of Numerical Restriction

Numerical restriction requires several difficult interrelated decisions. The first decision pertains to the number at which to set the ceiling. The second decision is whether to place a ceiling on all immigration or instead to have two immigration streams, one numerically limited, the other numerically unlimited. If the second decision is to have two streams, then the third decision pertains to the characteristics to be used for exempting one stream from the ceiling. The fourth decision, applying to all numerically limited immigrants, is how to choose from among a pool of applicants, for example, by a first-come/first-served rule or by lottery or by granting preferences or points. If the outcome of the fourth decision is to grant preferences or points, then the fifth decision pertains to the criteria to be used. A sixth decision is whether to add unused visas to the next year's supply of visas.

These are complicated matters, and it bears emphasizing that they engender much debate, as will be seen in the illustration in the section on Migration Restriction Regimes in the United States. The history of a country's immigration may be viewed as a history of asking and re-asking these questions, collected in Table 1.

**Table 1:** Apparatus for migration restriction: initial setup.

**A. Personal restriction**
Personal restriction grants or denies legal permanent residence (LPR) to individuals based on their personal characteristics.
1. Which characteristics confer or deny eligibility for legal permanent residence?
2. For qualitative characteristics, which categories confer or deny eligibility for LPR?
3. If origin country is one of the qualitative characteristics, how is country defined?
4. For quantitative characteristics, where is the line drawn between eligibility and ineligibility?

**B. Numerical restriction**
Numerical restriction limits the number admitted to legal permanent residence in a fiscal year.
1. What is the numerical ceiling?
2. Is a subset of individuals exempt from the numerical ceiling?
3. If a subset of individuals is exempt from the numerical ceiling, which characteristics generate the exemption?
4. Within the numerical ceiling, how are visa numbers allocated? By order of application, at random, or by personal characteristics?
5. If numerically limited visas are allocated based on personal characteristics, which characteristics matter and how are they prioritized?
6. If numerical ceilings are not reached in a given year, are unused visas added to the next year's pool of visas?

Moreover, as already hinted, both personal restriction and numerical restriction can be elaborated, further complicating setup of a migration restriction regime.

Personal restriction can be elaborated by noticing whether the characteristics constituting the criteria for restriction are fixed or alterable, a dimension crosscutting their qualitative or quantitative character. In general, one cannot change physical attributes or the things of the past. This set includes parental characteristics (such as parental religion), childhood characteristics (such as first language), and previous behaviors (such as previous membership in a political organization), as well as race and ancestry. However, other personal characteristics can be changed – e.g., schooling, occupation, bank account, religious affiliation – and a new language can be learned (Jasso, 2009b, p. 29, 34–36).

Similarly, numerical restriction differs importantly according to (1) whether there is a single numerically limited stream or dual streams (one numerically limited, the other not) and (2) whether the type of selection is first-come/first-served or random selection or preferential selection. Random selection is a “pure” numerical restriction. Preferential selection incorporates forms of personal restriction and thus is not a pure numerical restriction. First-come/first-served embeds additional processes, possibly including personal characteristics (e.g., in the urgency to flee and the resources to flee quickly).

Thus, the setup of migration restriction may be even more difficult and contentious. Still, the basic skeleton in Table 1 provides a foundation for analyzing migration restriction regimes.

### The Consequences of Migration Restriction

When the country restricting immigration is attractive, the immediate consequences of restriction are unauthorized migration (for both personal and numerical restriction) and visa-number backlogs (for numerical restriction)^1^.

As long as personal restrictions exist, ineligible people will enter the country in secret, or, if a temporary visit was permitted, remain, building a set of unauthorized residents.

Similarly, as long as numerical restrictions exist, eligible people will apply to immigrate, even when immigration is not possible for many years. And backlogs will accumulate. Moreover, some persons in the backlogs may enter/remain as unauthorized residents.

These immediate consequences spawn second-order consequences, in particular, policy devices to deal with them. The policy devices include enforcement measures as well as mechanisms for legalization and periodic clearing of backlogs^2^.

As well, restriction yields two interesting new metrics. For one way to assess the attractiveness of a country that restricts immigration is by the magnitude of unauthorized migration and visa-number backlogs. And one way to gauge the innovativeness and creativity of a country's government is by the policies it formulates to deal with unauthorized migration and visa backlogs. These policies are also a gauge of the country's humaneness and deepest values, as noted by Bhagwati and Rivera-Batiz (2013).

### Periodization by Migration Restriction Regime

Each of the four possible migration restriction regimes – fully unrestricted, personal restriction only, numerical restriction only, and fully restricted – has a distinctive apparatus and distinctive consequences, as discussed above. Accordingly, it may be useful to characterize the history of a country's immigration law by a periodization highlighting the four possible migration restriction regimes. For example, a country may or may not have a fully unrestricted migration regime in its history and/or it may or may not have an exclusively personal-restriction regime in its history, and so on. And the ordering of the regimes may be distinctive – and linked to the country's economic, social, and political features.

It may also happen that a country treats different parts of the world or different sets of countries differently, generating a somewhat more elaborate periodization. As will be seen in the section on Migration Restriction Regimes in the United States, the United States exemplifies this case, as for a period of over 40 years it had different rules for prospective immigrants from the Eastern Hemisphere and the Western Hemisphere.

## Migration Restriction Regimes in the Broader Social Science Context

Before proceeding to take a close look at migration restriction in the United States, it is useful to consider two broader social science matters. The first pertains to measurement, the second to substance, specifically the link between migration restriction and attitudes to immigration. Both embed a concern for fuller understanding of the determinants of migration restriction and the larger consequences beyond unauthorized migration and visa-number backlogs.

### Measurement of Migration Restriction Regimes

Across the social sciences, as appreciation has grown of the importance for human behavior of the social/economic/political environment, so, too, have efforts to measure relevant features of the environment and as well to understand their origins (see, inter alia, Weber's, 1892 pioneering examination of Polish workers in Germany; Thomas and Znaniecki's, 1927 pathbreaking work, The Polish Peasant in Europe and America; and Elder's foundational work on life course analysis, summarized in Elder et al., 2003). Some of these features are simple and straightforward to measure (e.g., length of the school year or length of the school day), others less simple but with a rich scholarly tradition (e.g., Gross Domestic Product), and still others quite challenging (e.g., migration policy regimes). Research organizations, policy institutes, and government offices have contributed data and insights, sometimes jointly, to advance measures of these important macro features.

Valuable exemplars include the Human Development Index (United Nations Development Programme, 2020), the Gender Gap Index (World Economic Forum, 2019), and the Human Capital Index (World Bank, 2019, 2020)^3^.

The field of migration has seen a major creative surge of efforts to conceptualize and measure migration policies (Bjerre et al., 2015; Filindra and Goodman, 2019), culminating in several large-scale projects: the Immigration Policies in Comparison (IMPIC) project covering 33 OECD countries in 1980-2020 (Helbling et al., 2017); the Determinants of International Migration Policy (DEMIG) project covering 45 countries in 1945-2014 (de Haas et al., 2015); the International Migration Policy and Law Analysis (IMPALA) project covering 9 countries in 1999-2008 (Beine et al., 2016); and the Migrant Integration Policy Index (MIPEX) project covering 52 countries in 5 continents, including all the EU member states and all the OECD countries, in 2007-2019 (Solano and Huddleston, 2020).

These projects have produced a rich literature that promises to substantially advance knowledge about international migration (Filindra and Goodman, 2019). By comparison, the framework introduced in this paper is modest. It has no intent to create an index or measure degree of restrictiveness. Moreover, its focus is largely on the internal structure of migration restriction regimes – their moving parts, conceptualized as two main types of restriction, personal restriction and numerical restriction – and while these moving parts affect the lives of migrants and all who enter migration systems (such as citizens sponsoring relatives and workers for immigration), it is also understood that the precise consequences depend not only on the migration restriction regime but also on the context. For example, the same policy may be thought exceedingly restrictive in a context of high demand and wonderfully generous in a context of low demand.

Yet both this framework for analyzing migration restriction regimes and the large migration policy projects arise from the same spirit, and both their points of convergence and their differences could yield useful synergies. To illustrate, both this framework and the larger policy projects encompass legal categories (such as legal permanent resident or citizen) and personal characteristics (such as language or religion); both cover long time spans; both implicitly or explicitly seek to understand both determinants and consequences of particular policies (considerations noted by Filindra and Goodman, 2019). Both are tools for understanding a wide range of domains – e.g., rights and responsibilities of non-citizens across the great diversity of legal categories. Indeed, description of a country's migration policy at a given point in time could benefit from both the policy indexes and the migration restriction framework, the latter classifying the regime as fully unrestricted, with personal restriction only, with numerical restriction only, or fully restricted. Additionally, it may be useful to go more deeply and distinguish within types of personal restriction and numerical restriction, as suggested in the section on The Apparatus of Migration Restriction above (e.g., distinguishing between fixed and alterable personal characteristics).

Finally, note a further immediately useful feature of the migration restriction framework proposed in this paper. Look again at Table 1. Each of the questions that the policymaker must address when setting up or revising a migration restriction regime is also an important feature of that regime. Examples include the presence or absence of personal restriction, of numerical ceilings, of dual numerically limited and numerically unlimited streams, and of the criteria embedded in them. Accordingly, any summary of a country's migration restriction regime would benefit from including the questions in Table 1.

Indeed, Table 1 leads immediately to the design of table shells for annual reports on migration systems, including both an overview table for all countries, in which the major features appear on rows and the countries in columns. One could then see at a glance, for a given year, whether each country's immigration law includes personal restriction or not and numerical restriction or not. Additional rows for each major feature could provide further information such as the numerical ceiling, if any, and the major personal characteristics used for personal restriction, if any. As well, this table could have a second panel, in which the rows represent persons of possible migration-relevant characteristics – including spouses, minor children, and parents of citizens and permanent residents, other relatives, and persons with a job offer in the country, as well as independent migrants with no familial relationship or prospective employment. In such a table, the reader could see at a glance which countries provide visas for specific kinds of individuals, for example, parents or siblings or independent migrants.

A second kind of table shell follows from the first. This would be a historical table for each country separately. Such a table would inform about changes over time in each country's migration restriction regime, reporting the start and end of particular provisions. One can envision an annual report whose first table is the worldwide table and this is followed by individual historical tables for each country.

A more detailed annual worldwide table could also display the number of persons admitted to permanent residence, separately by the number who are new arrivals and the number who are already in the country and adjusting their immigration status to permanent resident. Of course, the table could also display some of the consequences, such as the number in the visa-number backlogs for numerically limited visas and the estimate of unauthorized residents. The foregoing could also be incorporated into the historical country-specific tables.

### Migration Restriction Regimes and Attitudes Toward Immigration

Where do migration restriction regimes come from? Not from thin air. Migration restriction regimes reflect the attitudes and thinking of people and their countries. Even the briefest review of the literature on attitudes toward immigration suggests non-trivial variation across individuals, across countries, and over time (e.g., see for Europe, Heath et al., 2020; and for the United States, Smith and Edmonston, 1997, p. 389–393 and Waters and Pineau, 2015, p. 47–50, 147–148). As Heath et al. (2020, p. 475) observe, “Understanding what drives these… variations in public support for or opposition to immigration is therefore an issue of central importance for academics and policymakers alike.”

In general, the ensuing basic questions include, in classical terms, those emanating from the “functional prerequisites of a society” (Aberle et al., 1950), summarized in Jasso (1988b, p. 920): “How do societies recruit their members? How do groups decide membership criteria? What traits are deemed desirable in prospective members and what traits are not?”^4^ Other basic questions include philosophical questions about basic human rights and about how to allocate scarce benefits, as well as empirical questions about whether immigration policies awaken the sense of justice.

A subset of ethical questions may pertain to countries with particular historical origins. For example, discussing attitudes toward immigration in the United States, Weissbrodt et al. (2017, p. 52–53) observe, “Given the U.S. tradition as a country of immigrants, it is difficult to comprehend how current citizens – almost all of whom have benefited from immigration – can claim any right to exclude future immigrants.”

With respect to whether migration restriction awakens the sense of justice, justice theory offers three ways to think about this question. First, there is little doubt that migration restriction awakens the sense of justice, at least in people who experience the sense of justice, that is, all but the justice-oblivious who are thought to be a small set (Jasso, 2017, p. 612–613)^5^. Second, however, given the inherent subjectivity of the sense of justice – enshrined in the Hatfield-Friedman Principle, “Justice is in the eye of the beholder” (Walster et al., 1973, p. 152, 1976, p. 4; Friedman, 1977) – there is no a priori conclusion that any element of migration policy is just or unjust or that one policy might be more, or less, unjust than another. Third, justice theory yields a range of testable implications deduced from the basic postulates in the theory. The implications cover the behavior of migrants as well as people and policymakers in both origin and destination countries (Jasso, 1986, 1988a, 1996). Like all the implications of justice theory they are ceteris paribus implications, because justice is thought to be only one of the basic forces governing behavior (Jasso, 2008). Here is a sampling^6^:

1. Societies in which immigration and population growth are welcomed must be societies in which people value at least one cardinal good, such as wealth.
2. If the origin and destination countries have the same average wealth, they cannot both favor or both oppose the migration; they can only both be indifferent to it.
3. A necessary condition for the origin and destination countries to both want the migration is that they be unequal in average wealth.
4. Two conditions jointly necessary and sufficient for the origin and destination countries to both want the migration are that migration be from a poor country to a rich country and that the migrant lie above the mean of the origin country and below the mean of the destination country.
5. Two conditions jointly necessary and sufficient for the origin and destination countries to both oppose the migration are that migration be from a rich country to a poor country and that the migrant lie below the mean of the origin country and above the mean of the destination country.

There is ample evidence that people often have diametrically opposed ideas about what is just in the world of migration policy. These ideas come to be formalized in political party platforms and non-governmental advocacy groups^7^. Notwithstanding the subjectivity of ideas of justice, it is possible that a general justice principle could emerge via sustained theoretical analysis. For example, while one would think that, given the Hatfield-Friedman Principle, ideas of what constitutes “the just society” would differ among persons, deductive reasoning yields the surprising prediction that “The just society has a mixed government; distribution of benefits is by the many, and distribution of burdens is by the few”^8^. Thus, it remains possible that migration too would be surprised by a general justice principle. Such a general justice principle would transcend the competing ideas of what is just, calming what might appear to be an inherent instability of migration restriction regimes. Indeed, the multi-country empirical work reported and discussed in Heath et al. (2020) and the references cited therein, such as Davidov et al. (2020), together with single-country studies such as Jasso (1988b), Diehl and Steinmann (2012a,b), and Diehl et al. (2018), may yield the components for a new general principle of justice about migration.

As for migration and human rights, there is a curious asymmetry. Human rights documents, such as the Universal Declaration of Human Rights (UDHR) adopted by the United Nations General Assembly in Paris on 10 December 1948^9^, protect the right to leave one's country but leave unaddressed the corresponding right to enter another country. The conversation between President Jimmy Carter of the United States and Deputy Premier Deng Xiaoping of China during Deputy Premier Deng's state visit to Washington in late January 1979, after the two countries had normalized relations on the 1st of January, is illuminating (Foster, 2015):

[When the United States] established diplomatic relations in 1979, the United States considered whether the Jackson-Vanik Amendments to the Trade Act of 1974, which required the US to impose trade restrictions on any country that restricted emigration, applied to China as it did to the Soviet Union. However, during the historic 1979 visit of paramount leader Deng Xiaoping to the United States, when he was asked by then President Jimmy Carter about Chinese restrictions on outbound emigration, Deng Xiaoping's reported response was “How many millions do you want?” Thereafter, the United States showed little or no interest in Chinese emigration policy.

Yet symmetry is much on the mind of Pope Francis (2020) who proposes “safe corridors” for migrants to move from one country to another.

## Migration Restriction Regimes in the United States

To begin, consider restriction on admission to legal permanent residence (LPR), popularly known as getting a “green card” – considering the types of restriction, the apparatus for restriction, and the consequences of restriction. This leads naturally to a restriction-focused periodization of U.S. immigration history^10^.

**Figure 1:**
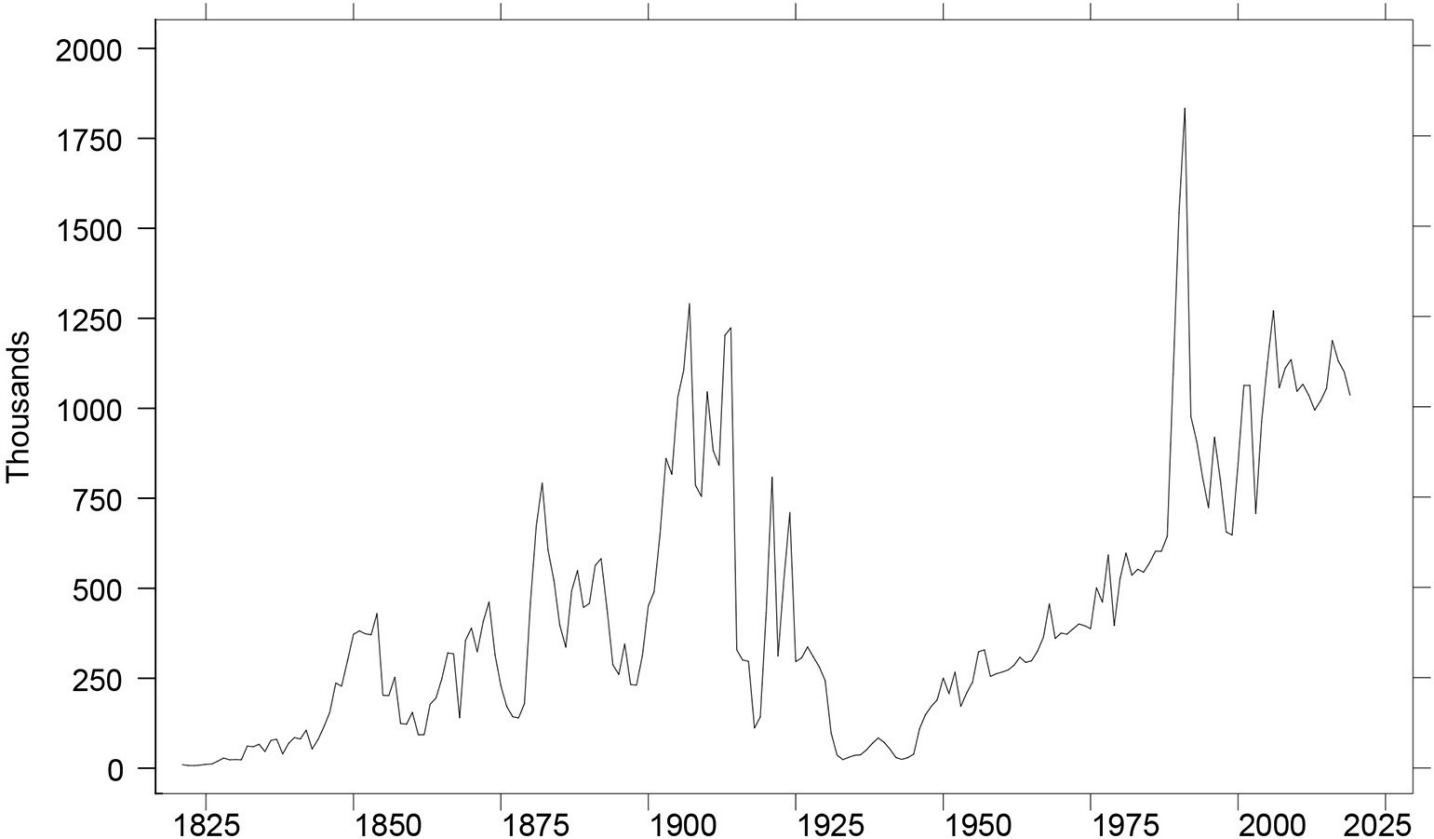
Immigration to the United States: 1820-2019. Annual totals represent the number of persons admitted to legal permanent residence (U.S. Department of Homeland Security, 2019, Table 1).

To set the stage, Figure 1 depicts annual admissions to legal permanent residence in the United States since 1820. Annual totals are from Table 1 of the 2019 Yearbook of Immigration Statistics, the most recent annual report of the U.S. Department of Homeland Security (DHS). The graph shows the spike in 1991 and surrounding years due to persons acquiring LPR via the legalization provisions of the Immigration Reform and Control Act of 1986 (IRCA). This graph is probably the best-known graph in the entire field of U.S. immigration, published widely in the annual reports of the U.S. Immigration and Naturalization Service and the Annual Flow Reports of the U.S. Department of Homeland Security^11^.

Previewing fuller description below, restriction is of two kinds, personal (noticing characteristics of persons, inclusive of national origin) and numerical (placing a ceiling on all or a subset of immigrants). Both types of restriction require an apparatus (what characteristics to favor or bar, whether to exempt some visa applicants from a ceiling and by what criteria, what ceiling to place on numerically limited immigration, etc.). Both types of restriction engender unauthorized immigration; numerical restriction also engenders backlogs. The twin consequences of unauthorized and backlogs in turn lead to new policy devices, such as mechanisms for enforcement, legalization, and periodic clearing of backlogs.

The elements of personal and numerical restriction, as well as the policy devices to deal with consequences of restriction, are codified in U.S. law and policy. For example, the elements of personal restriction appear in laws that establish the grounds of inadmissibility, distinguishing between admissibility for temporary or permanent residence and providing exceptions as well as waivers. Important sources for studying U.S. migration restriction, besides original pieces of legislation, court cases, and executive actions, include the United States Code (USC), Title 8, which is a compilation of all legislation on immigration, and the Code of Federal Regulations (CFR), Title 8, which is a compilation of all immigration procedures. Both 8 USC and 8 CFR are titled “Aliens and Nationality.” Also indispensable is the Policy Manual of the U.S. Citizenship and Immigration Services. The USCIS Policy Manual, still under construction, is the successor to the Adjudicator's Field Manual; these provide the basics of immigration law and policy as guidance to immigration officers. They also provide links to 8 USC and 8 CFR^12^.

As well, the two types of restriction, combined with the geographic and historical distinction between the Eastern and Western Hemispheres, lead to a periodization of U.S. immigration history to date into Four Immigration Eras, beginning with an era of neither personal nor numerical restriction (to 1874), continuing to a Second Immigration Era, characterized by personal restriction only (to 1920), and a Third Immigration Era, with numerical restriction on the Eastern Hemisphere. Finally, the 1965 Immigration Act, by extending numerical restriction from the Eastern Hemisphere to the whole world, ushered in the Fourth Immigration Era.

This section ends with a close look at the 1965 Act, an assessment from the migration restriction perspective and a look ahead, asking whether there might be a Fifth Immigration Era and how it might look.

### Elements of Migration Restriction in the United States

#### Types of Migration Restriction in the United States

As noted and as will be described in fuller detail, during its history, the United States has had a period of no restriction, a period of personal restriction only, a period of personal restriction combined with numerical restriction on one hemisphere, and a period of personal restriction combined with worldwide numerical restriction. It has never had numerical restriction without personal restriction.

#### Apparatus of Migration Restriction in the United States

##### Elements of Personal Restriction

As discussed above, questions of personal restriction are not easy, and they have been and continue to be vigorously debated by both legislators and citizenry. For example, in 1980 when the U.S. Select Commission on Immigration and Refugee Policy was exploring the possibility of a point system for the selection of immigrants, the Commission's professional staff was surveyed using a sophisticated factorial survey method that made it possible to estimate the point system each person would favor^13^. Notably, no two estimated point systems were alike (Jasso, 1988b, p. 928–929). For example, only two characteristics were signed the same way by all staff members, having a job offer and having a sibling who is a U.S. citizen, both increasing the applicant's desirability; however, staff members differed with respect to which characteristic would provide more points. Staff members disagreed on whether to grant more points to men or to women and on whether to grant points for knowledge of English. Similarly, while staff members were unambiguously attentive to continent of birth, net of the percentage of visas received by the prospective immigrant's co-nationals in the last 5 years, they disagreed on the ordering, providing points in distinctive ways. For example, one staff member gave applicants from Africa 53 more points than applicants from Latin America, while giving 24 points for having a citizen sibling and 23 points for having a job offer; thus, for that staff member, an applicant from Latin America with both a citizen sibling and a job offer would get a lower score than an applicant from Africa with neither^14^.

##### Elements of Numerical Restriction

As discussed above, questions of numerical restriction require several interrelated decisions (as shown in Table 1). These are visible in a country's history, for example, in the summaries of briefings and consultations in the reports of the U.S. Select Commission on Immigration and Refugee Policy. Indeed, the history of U.S. immigration law can be viewed as a history of asking and re-asking these questions, continually modifying the answers – for example, exempting professors from the numerical ceiling in one law and subsequently moving them to the numerically-limited stream, or placing husbands of U.S. citizens in the numerically-limited stream and subsequently moving them to the exempt stream^15^.

##### Vocabulary of Migration Restriction

Migration restriction requires a special vocabulary. Two words in the vocabulary pre-exist migration restriction: alien and immigrant. Derived from Latin (“stranger” or “foreigner”) and inherited from English common law, the word alien was first used in 1798 in the Alien and Sedition Acts. It is defined in U.S. immigration law as “any person not a citizen or national of the United States,” and the USCIS Glossary adds, “‘Foreign national’ is a synonym and used outside of statutes when referring to non-citizens of the U.S.” The word immigrant, also derived from Latin, originally referred to anyone moving to the United States. But numerical restriction would give it a new and restricted meaning.

If numerical restriction classifies aliens into distinct legal categories, then special words are needed to refer to these distinct situations. The Immigration Act of 1924, building on the basic ideas in the Emergency Quota Act of 1921, provided a new definition of immigrant – in essence a precursor to the contemporary permanent resident – excluding diplomats, tourists, aliens in transit, merchant seamen, and treaty traders from the set of immigrants and giving these a new name: non-immigrant (Parker, 1924; U.S. Public Law, 68-139, 1924).

Next, the Immigration Act of 1924 distinguished two kinds of immigrants, non-quota and quota. The 1924 Act's non-quota and quota immigrant classifications are the precursors, respectively, of the contemporary numerically unlimited immigrant and numerically limited immigrant categories. The non-quota class included wives and unmarried children under 18 of U.S. citizens residing in the United States, returning residents, natives of the Western Hemisphere, ministers and professors and their wives and unmarried children under 18, and students at least 15 years of age entering an approved course of study (Parker, 1924; U.S. Public Law, 68-139, 1924).

The Act defines quota immigrants as immigrants who are not non-quota immigrants. Quota immigrants were subject to numerical restriction based on national origins plus a system of preferences. For example, preference would be given to the unmarried children under 21 years of age, parents, and spouses of U.S. citizens age 21 or over, and to agricultural workers and their wives and dependent children under 16 years of age (Parker, 1924; U.S. Public Law, 68-139, 1924).

Finally, the 1924 Act introduced the word visa. Also based on Latin (for “to see”), a visa certified that a prospective immigrant's application had been seen and approved by a consular officer abroad (Parker, 1924; U.S. Public Law, 68-139, 1924). Indeed, the Act uses both noun and verb, referring to “an immigration visa which shall consist of one copy of the application…, visaed by such consular officer” (in the section on Migration Restriction Regimes)^16^.

Of course, words are living things. They come and go, and their meaning changes. Perhaps due to film and television, the word alien became associated with extraterrestrial life forms (some friendly, some not), and increasingly the synonym “foreign national” was used in immigration discourse (as in the USCIS Glossary, noted above).

But words are also vulnerable to conscription by political wordsmiths. On 8 October 2019 the USCIS Policy Manual published a “Technical Update” subtitled “Replacing the Term ‘Foreign National’.” The Update states:

This technical update replaces all instances of the term “foreign national” with “alien” throughout the Policy Manual as used to refer to a person who meets the definition provided in INA 101(a)(3) [“any person not a citizen or national of the United States”].

Nothing was safe from the new deployment, not even the venerable annual reports published by the Office of Immigration Statistics (OIS) at DHS, which form the statistical foundation for much immigration research – the Annual Flow Reports and the annual Population Estimates^17^. The opening sentence of the “Annual Flow Report” on legal permanent residents went from

Immigration law defines a lawful permanent resident (LPR) or “green card” recipient as *a person* [italics added] who has been granted “the status of having been lawfully accorded the privilege of residing permanently in the United States as an immigrant in accordance with the immigration laws, such status not having changed.

for the 2018 cohort (Baugh, 2019) to

Immigration law defines a lawful permanent resident (LPR) or “green card” recipient as *an alien* [italics added] who has been granted “the status of having been lawfully accorded the privilege of residing permanently in the United States as an immigrant in accordance with the immigration laws, such status not having changed.

for the 2019 cohort (Baugh, 2020). A footnote was added to “alien” providing the definition in the Immigration and Nationality Act (and in the USCIS Glossary), namely, “An alien is any person not a citizen or national of the United States.”

Similarly, in the Population Estimates reports for LPRs, the phrase “unauthorized immigrants” (Baker, 2019a, p. 2) was changed to “illegal aliens” (Baker, 2019b, p. 1). The change in the Population Estimates reports for the unauthorized was more extensive, changing not only the opening sentence but also the title. The opening sentence went from

This report provides estimates of the size of the *unauthorized immigrant* [italics added] population residing in the United States as of January 2014 by period of entry, region and country of origin, state of residence, age, and sex.

for the January 2014 estimate (Baker, 2017) to

This report provides estimates of the size of the *illegal alien* [italics added] population residing in the United States as of January 2015 by period of entry, region and country of origin, state of residence, age, and sex.

for the January 2015 estimate (Baker, 2018). A footnote was added to “illegal alien” with the following text:

The Department of Homeland Security refers to foreign-born non-citizens unlawfully present in the United States as “illegal aliens.” Previous versions of this report used the term “unauthorized immigrants” to refer to this population.

Further adventures in this “war of the words” no doubt await (Shear and Jordan, 2021).

#### The Consequences of Migration Restriction in the United States

As discussed above, when the country restricting immigration is attractive, the immediate consequences of restriction are unauthorized migration (for both personal and numerical restriction) and visa backlogs (for numerical restriction). The United States provides a prime example that the immediate consequences of restriction are unauthorized migration and visa backlogs. As noted by Masanz (1980, p. 33), “According to the Immigration Bureau [in the Annual Reports of 1922 and 1923], the increase in the various restrictions on alien entry into the United States was accompanied by an increase in the number of surreptitious entries and, eventually, in the establishment of a thriving smuggling industry.” The 1922 Annual Report (U.S. Commissioner General of Immigration, 1922) specifically mentions that prospective immigrants desiring “to evade the restrictions of the ‘quota’ act have proceeded to both Canada and Mexico in large numbers, and it is these who have endeavored, and are endeavoring, to gain admission by stealth, usually with the aid of hired smugglers” (quoted in Masanz, 1980, p. 3). The official INS history of immigration (U.S. Immigration and Naturalization Service, 1979-2001, p. 11) states, “An unintended result of the quota system's limits on immigration was a great rise in illegal immigration by 1923.” Ninety years later, Bhagwati and Rivera-Batiz (2013, p. 12) observe, “as long as immigration restrictions exist, people will continue to enter the United States illegally,” and, one might add, overstay legal visas and work without authorization.

Similarly, as long as numerical restrictions exist, eligible people will apply to immigrate, even when immigration is not possible for many years. And backlogs will accumulate. Virtually every primary and secondary source on the history of U.S. immigration since 1921 includes some mention of backlogs. For example, the official U.S. Immigration and Naturalization Service (1991, p. 21) history of immigration refers to “quota backlogs [becoming] too large” in the 1950s, and Vialet (1979, p. 62–63), in the history of immigration law prepared for the use of the newly established U.S. Select Commission on Immigration and Refugee Policy, describes the rapid development of a Western Hemisphere backlog after imposition of the numerical ceiling in 1968.

These immediate consequences spawn second-order consequences, in particular, policy devices to deal with them. The policy devices include enforcement measures as well as mechanisms for legalization and periodic clearing of backlogs.

Enforcement measures include deportation and border measures. It is no accident that, as noted by Masanz (1980), the Border Patrol was established as part of the provisions of the Act of 28 May 1924 – 2 days after the Immigration Act of 1924.

Another policy device is legalization. Again, it is no accident that the registry provision of U.S. law was established within 5 years of the Immigration Act of 1924, via the Registry Act of March 2, 1929. Under this provision, a record of admission is created for aliens whose record of admission cannot be found and who meet certain criteria, including residence in the United States since before a certain date. In 1929 that date was set in 1924. Subsequently it was moved several times, and currently stands at 1 January 1972. Table 2 reports the date required for inception of residence by each law since the registry provision was established.

**Table 2:** Legalization of unauthorized: U.S. immigration registry law.

**Year of Act**	**Entry date**	**Years in U.S. required**	
		**Shortest**	**Longest**
1929	1 July 1924	5	15
1939	3 June 1921	18	19
1940	1 July 1924	16	34
1958	28 June 1940	18	25
1965	30 June 1948	17	38
1986	1 January 1972	14	49

Although the registry provision was ostensibly intended for persons who wanted to naturalize but did not have or could not locate the requisite record of admission, and deportable aliens were not explicitly mentioned until legislation in 1958, it may have been used as a legalization tool (Bruno, 2001; Wasem, 2010). Indeed, a 1936 description in the Statistical Abstract (U.S. Department of Commerce, 1936, p. 104) states that the registry legislation “legalizes permanent residence in the United States.” And the website of the U.S. Citizenship and Immigration Services (USCIS) describes the registry files for the period March 2, 1929, to March 31, 1944, available for genealogical searches, as documenting “the first ‘legalization program’ authorized by Congress”^18^.

Other policy devices include temporary legalization. The 1952 Act granted the Attorney General parole authority, whereby persons otherwise inadmissible can be granted temporary entry on humanitarian grounds (Wasem, 2010).

Similarly, the 1990 Act introduced a new way to allow unauthorized migrants to remain in the United States temporarily, authorizing the Attorney General to grant temporary protected status (TPS) to undocumented alien nationals of designated countries undergoing armed conflict, natural disasters, epidemics, or other conditions which temporarily prevent the migrants' safe return (U.S. Immigration and Naturalization Service, 1979-2001, Appendix 1–20).

As for backlogs, virtually all discussions of immigration legislation include earnest discussions about how to structure eligibility for LPR so that backlogs do not accumulate. Additionally, modifying the basic apparatus for restriction can clear backlogs. For example, the U.S. Select Commission Staff Report of 1981 notes that the Senate Committee charged with reviewing the immigration system in 1947-1950 considered moving parents of U.S. citizens and husbands of U.S. citizens (regardless of the date of the marriage) from the numerically-limited stream to the numerically-unlimited stream, moves which would clear the parent backlog (then facing a wait of 7 to 8 years) and reduce the backlogs for Greece, Portugal, Romania, Spain, and Turkey (U.S. Select Commission on Immigration Refugee Policy, 1981, p. 313).

Moving a subset to the numerically unlimited stream would of course clear the backlog for that subset. However, given the high demand for permanent visas, it is unlikely that any modification of the criteria for numerically-limited immigration would prevent backlogs.

This discussion has focused on the consequences of migration restriction for the migration restriction regime. Of course, there are also consequences for all actors and countries in the migration process – from talent lost or delayed for the destination country and remittances lost or delayed for the origin country to a range of effects on the life chances of individuals and the stratification structures of both origin and destination countries (Jasso, 2011).

### A Periodization of U.S. Immigration History Based on Migration Restriction

Before 1875 immigration to the United States was largely unrestricted, although there was substantial restriction on citizenship and naturalization. For example, the Naturalization Act of 1790 limited naturalization to “free white persons” and there was legislation on such matters as the residency period required for naturalization and the link between gender, marriage, and naturalization (Smith, 1998). But immigration *per se* was largely unrestricted. Thus, the period 1789-1874 was a no-restriction era and may be considered the First Immigration Era, a Pre-Restriction Era.

The Immigration Act of 1875 marks the start of personal restriction on U.S. immigration and thus may be considered the start of the Second Immigration Era. It prohibited for the first time the entry of persons considered undesirable, barring prostitutes and convicts. It would be followed by a long string of laws, noticing a large variety of personal characteristics, conditions, and behavior, starting with race in 1882 (Chinese Exclusion Act) and accumulating a rapidly growing list of inadmissibles, such as paupers, contract laborers, persons with certain contagious diseases, polygamists, anarchists, feeble-minded persons, unaccompanied minors, illiterates, and other Asians.

But personal restriction did not mitigate the growing discontent with immigration, and 1921 brought the Emergency Quota Act of May 19, 1921, introduced above, placing a ceiling of 357,000 on immigration from the Eastern Hemisphere, the first numerical restriction on U.S. immigration and thus marking the start of the Third Immigration Era. However, a subset was exempt from the numerical ceiling, including actors, singers, professors, and ministers. The Quota Law, which was temporary “emergency” legislation, was quickly extended for 2 years (with an amendment to increase from 1 to 5 years the requisite period of residence in the Western Hemisphere to qualify for exemption from the ceiling), then followed by the Immigration Act of 1924, which revised and codified all elements of the apparatus for restriction. It reduced the ceiling to 164,000, modified the criteria for exemption from the ceiling, and modified the national origins formula and introduced a system of preferences for the numerically limited stream. It also introduced the provision that aliens ineligible for citizenship could not be admitted to legal permanent residence, as discussed by Parker (1925).

There followed a long string of new laws, including the 1924 law establishing the Border Patrol and the 1929 law establishing the registry provision, discussed above, as well as laws modifying the apparatus for restriction (e.g., a 1932 law exempting from the numerical limit the husbands of U.S. citizens, provided that the marriage occurred prior to issuance of the visa and prior to July 1, 1932)^19^.

World War II brought new concerns and new legislation, mostly for security but also dismantling some of the elements of restriction – for example, two 1940 laws extending naturalization to military personnel regardless of race and permitting the naturalization of indigenous races of the Western Hemisphere, a 1943 law extending naturalization to Chinese persons and persons of Chinese descent (now that China was a close wartime ally of the United States), a 1946 law which gave non-quota status to the Chinese wives of U.S. citizens, a 1948 law extending naturalization to Filipino persons or persons of Filipino descent and to persons of races indigenous to India, and a 1950 law providing non-quota status to the spouses and minor children of members of the American armed forces, regardless of race (provided that the marriage occurred before 19 March 1952), as well as the landmark Immigration and Nationality Act of 1952, which eliminated all racial and gender bars to naturalization but, over President Truman's veto, retained the national origins formula for the numerically limited stream.

Other laws provided for clearing backlogs, for example, a 1962 law giving non-quota visas to certain applicants for fourth preference (brothers, sisters, and children of citizens) and first preference visas (special occupational skills). Notably, not long after President John F. Kennedy issued the groundbreaking Executive Order 10925 prohibiting discrimination in government employment and employment by government contractors on the basis of “race, creed, color, or national origin” (6 March 1961), legislation was enacted eliminating the requirement that visa applicants provide their race (26 September 1961).

Pressure mounted for elimination of the national origins quotas, and after 13 years Congress passed the Immigration and Nationality Act of 1965 (also known as the Hart-Celler Act), abolishing the national origins quotas. The price was extending the numerical ceiling to the Western Hemisphere, thus ushering in the Fourth Immigration Era, as shown in Table 3.

**Table 3:** Four immigration eras in the United States, classified by type of migration restriction and hemisphere.

**Type of restriction**	**Eastern hemisphere**	**Western hemisphere**
**1. First Immigration Era: 1789-1984**		
Personal	No	No
Numerical	No	No
**2. Second Immigration Era: 1875-1920**		
Personal	Yes	Yes
Numerical	No	No
**3. Third Immigration Era: 1921-1964**		
Personal	Yes	Yes
Numerical	Yes	No
**4. Fourth Immigration Era: 1965-**		
Personal	Yes	Yes
Numerical	Yes	Yes

The 1965 Act also modified the apparatus for restriction, inclusive of the numerical ceiling, the criteria for exemption from the numerical ceilings, and the criteria for prioritization within the numerically limited stream^20^.

**Figure 2:**
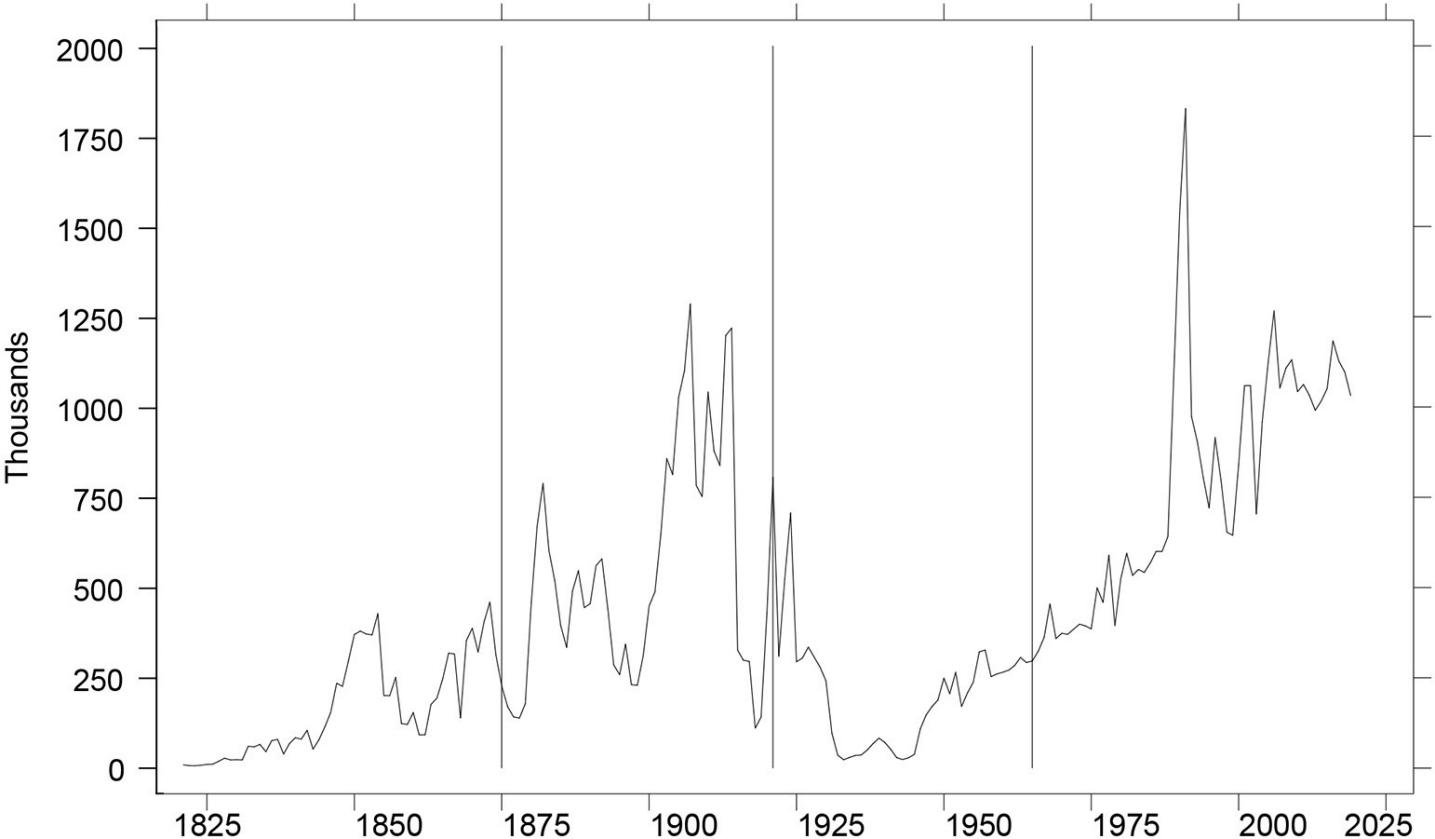
Immigration to the United States: Four Immigration Eras, 1820-2019. Vertical lines at 1875, 1921, and 1965, marking the start of the Second through Fourth Immigration Eras.

Figure 2 provides a view of the restriction-focused periodization of U.S. immigration history. It includes vertical lines at 1875, 1921, and 1965, marking the start of the Second through Fourth Immigration Eras^21^.

### A Close Look at the 1965 Act

The migration restriction perspective enables focused assessment of each piece of legislation. To illustrate, consider the 1965 Act. Of course, each Immigration Era and, within each Era, each piece of legislation merit sustained assessment. For example, the Immigration Reform and Control Act (IRCA) of 1986, the “smaller” Immigration Marriage Fraud Amendments Act of 1986, the Immigration Act of 1990, and the Illegal Immigration and Immigrant Responsibility Act (IIRIRA) of 1996 produced far-reaching changes to both personal criteria and numerical criteria for LPR admission as well as to associated procedures and requirements. Here the focus is on the 1965 Act, in part because it is “boundary” legislation, ushering in the current era, the Fourth Immigration Era, in part because two of its provisions are central to the history of U.S. immigration law – abolition of national origins quotas and the end of distinctive treatment for the Eastern and Western Hemispheres.

What did the 1965 Act accomplish? Look at Table 1. The Act provided modified answers to the questions underlying the apparatus of numerical restriction. It provided a new ceiling for numerically-limited immigration (at first separate ceilings for the two Hemispheres, subsequently a single worldwide ceiling starting in 1977). It modified the criteria for numerically-unlimited immigration, moving parents of U.S. citizens from the second preference to the unlimited stream. It altered the preferences for the numerically-limited stream (at first only in the Eastern Hemisphere, extended in 1976 to the Western Hemisphere), for example, moving employment immigrants from first preference to third and sixth preference (skilled and unskilled, respectively) and moving spouses of legal permanent residents from third preference to second preference.

What about unauthorized migration and visa backlogs? Whatever the modifications of the 1965 Act to the apparatus for restriction, they did not prevent either unauthorized migration or visa backlogs. Both grew quickly – unauthorized migration to about 3.5 to 5.0 million and backlogs to over a million by 1980, as noted in the staff report of the U.S. Select Commission on Immigration Refugee Policy (1981, p. 377, 482).

And what about policy devices for dealing with unauthorized migration and visa backlogs? The 1965 Act did not invent the registry provision (1929) or the Attorney General's parole authority (1952) or temporary protected status (1990). It did not invent the Diversity Visa Program (1990), which has made it possible for persons from all over the world to come legally to the United States, competing by lottery for 50,000 visas annually.

Indeed, within less than a decade and a half, pressure would mount to review immigration law, and Senator Edward M. Kennedy, the great champion of the 1965 Act, would write, in the Introduction to the history of immigration law prepared in 1979 for use by the newly established U.S. Select Commission on Immigration and Refugee Policy (Vialet, 1979, p. 1):

The current Immigration and Nationality Act is a generation out of date. It is out of touch with the times, and inadequate to meet modern needs. It was enacted in 1952 over President Truman's veto, at the depth of the cold war and the restrictionist atmosphere of that era. It was flawed from the beginning with discriminatory and anti-alien provisions. Some of the more blatantly racist and objectionable sections – such as the national origins quota system and the Asia-Pacific Triangle provisions – were repealed in 1965.

But not much else has changed. The 1952 Act is still the basic statute governing immigration. But after more than a quarter century, its provisions and administrative procedures are seriously inadequate. And, until the last 2 years, little has also been done to strengthen the role of the Immigration and Naturalization Service in implementing the law.

Without doubt the greatest contribution of the 1965 Act was abolition of the national origins quotas – a strong and proud statement that the United States pays no attention to race and nationality.

In years to come, however, a case may be made that the imposition of numerical restriction on the Western Hemisphere dealt a mortal blow to cherished ideas about the New World, about countries which started as colonies of European powers and threw off the bonds, about countries with a weaker link between ancestry and nationality, about Good Neighbors. Perhaps it was valuable to affirm that not only American Indians born in Canada can move freely to the United States (as per the John Jay Treaty of 1794), and obtain LPR, but that so too could all natives of the Americas.

### Prospects for a Fifth Immigration Era

Might there be a Fifth Immigration Era in the United States? What would it look like? First, a Fifth Era could reprise one of the three Eras before the current Fourth Era. That is, U.S. immigration law could return to the fully unrestricted migration regime, as the First Immigration Era. Or it could return to an exclusively personal-restriction regime, as the Second Immigration Era. Or it could return to worldwide personal restriction but numerical restriction only on prospective immigrants from the Eastern Hemisphere, as the Third Immigration Era.

Second, a Fifth Era could continue to disregard Hemisphere but, unlike the Second or Fourth Eras, it could institute an exclusively numerical-restriction regime, with no personal restriction at all.

Third, a Fifth Era could revive the Hemisphere distinction and institute one of several variants: (1) exclusive personal restriction on the Western Hemisphere and exclusive numerical restriction on the Eastern Hemisphere; (2) exclusive personal restriction on the Eastern Hemisphere and exclusive numerical restriction on the Western Hemisphere; (3) a fully unrestricted regime for the Western Hemisphere and a fully restricted regime for the Eastern Hemisphere; (4) a fully unrestricted regime for the Eastern Hemisphere and a fully restricted regime for the Western Hemisphere.

One can imagine other possibilities for a Fifth Era. For example, a new migration restriction regime could retain the current disregard for Hemisphere but notice something entirely new such as planetary provenance. The fully restricted regime of the Fourth Era could continue for earthlings but extra-terrestrials would face neither personal nor numerical restriction.

For the time being, however, it would seem that the Fourth Immigration Era will continue. Of course, and compatible with the Fourth Immigration Era, there could be many changes in the personal criteria used to favor or bar immigrants and many changes in the numerical ceilings, as well as changes in immigration procedures.

Indeed, a new change has begun almost imperceptibly. It was noted above that soon after President John F. Kennedy's Executive Order 10925 prohibiting discrimination in government employment and employment by government contractors on the basis of “race, creed, color, or national origin” (6 March 1961), legislation was enacted eliminating the requirement that visa applicants provide their race (26 September 1961). Recently, questions on race and Hispanic origin have begun to appear in the USCIS forms used by immigrant applicants and their sponsors, for example, in the basic form used by sponsors of relatives (Form I-130, Petition for Alien Relative), in the form used by applicants for legal permanent residence who are already in the United States (Form I-485, Application to Register Permanent Residence or Adjust Status), in the form used to file for removal of conditionality restrictions by immigrants who qualified for legal permanent residence on the basis of a marriage of less than 2-years' duration (Form I-751, Petition to Remove Conditions on Residence), and in the form used to file for naturalization (Form N-400. Application for Naturalization).

## Afterword

### Toward Possible New Creative Policy Devices

Perhaps the 1965 Act has been held to an impossible standard. Perhaps the apparatus of restriction does not admit of more creative innovations. Perhaps neither do the policy devices to deal with restriction. Past history suggests two incantations (visible in Figures 1, 2), and they are not happy to contemplate: economic crisis and war.

Moreover, with the exception of lotteries and parole authority and temporary protected status, creative and happy policy devices are scarce. It is telling that although probably the entire country agrees that the “immigration system is broken,” there is pervasive disagreement about what precisely is broken. To some, what is broken is one or another element of the apparatus for restriction – ceiling too high or too low, persons included or excluded from the numerically exempt categories, and so on. To others, what is broken is one or another of the consequences of restriction – too much unauthorized migration (currently estimated at about eleven million), too large backlogs (currently at 3,978,487 approved and waiting in line for the approximately 366 thousand preference category visas given annually, as reported by the U.S. Department of State, 2020)^22^. To still others, what is broken pertains only to administrative matters – too many processing delays or too high fees associated with the immigration application process.

Interestingly, it seems to be generally accepted that, as the U.S. Select Commission on Immigration Refugee Policy (1981, p. 384) observed almost 40 years ago, “the United States can never return to a policy of open migration or the massive migrations of the nineteenth and early twentieth centuries…” Numerical restrictions appear here to stay, albeit, as Zolberg (1983, p. 13) put it, “with no precise rationale offered for them other than their self-evident necessity.”

Still, there may be a few policy devices useful all around. Consider unauthorized migration. Immigration researchers believe that a portion of the set of unauthorized consists of persons in the backlogs, who rather than wait in the origin country for the visa to become available, wait in the United States. Some were in the U.S. with temporary visas and have U.S.-born children. Like all parents, they want the best for their children, and in this case there is a happy coincidence of interests among the parents and the larger citizenry, for the longer and deeper the experience of Americanization, the more fluent in English and the more productive Americans the children will be. The challenge is how to provide this experience of Americanization without increasing unauthorized migration. The possible numbers are not trivial. As noted above, the backlogs are massive – almost four million persons waiting for numerically-limited visas granted at the rate of about 366,000 a year, suggesting, on average, a wait of over 10 years, not counting a further period for administrative processing.

One approach is to make unauthorized residence in the United States less urgent for prospective immigrants in the backlogs by utilizing the vast American network around the world to provide advance training in English as well as some modicum of socialization into American life. The American network around the world has many components, emanating from both the public and private sector, that could be enlisted in this effort. Consider four: The first component of the U.S. global network that could be used in this effort consists of the schools operated by the Department of Defense, via the Department of Defense Education Activity (DoDEA) for the children of military personnel stationed at military installations abroad. The second component of the international U.S. network consists of the American-sponsored overseas schools assisted by the Department of State via the Office of Overseas Schools. The third component includes all the programs of the Bureau of Educational and Cultural Affairs, including the Office of English Language Programs. The fourth component is the growing network of American universities with branches abroad.

It would be useful to assess the possibilities for enlisting the substantial American presence abroad in the service of providing English language training and early Americanization for future LPRs around the world who are in the visa-number backlogs waiting for numerically-limited visas, thus tamping down the urgency to take up residence in the United States.

Another approach would bypass countries and their immigration laws. Suppose that migration restriction regimes are indeed inherently unstable – with fundamental disagreements on their moving parts and continual discussions of the questions in Table 1—and that a general justice principle remains elusive, unlike the case of the just society noted earlier or economic inequality, where it is possible to say with some albeit limited confidence that “inequality in the distribution of a good is a bad” (Jasso, 2017). Suppose further that natural disasters and political upheavals continue to generate large numbers of displaced persons urgently in need of refuge. Finally, suppose that at least one important sector of society – research and education – depends on free exchange of ideas and unrestricted travel to conferences. Then it might be possible, with international cooperation and generous philanthropy, to establish a network of conference centers around the world, governed by an international consortium, on land or islands contributed by countries or new artificial islands, staffed by migrants and refugees, providing not only all the amenities of a high-functioning conference center but also training for its staff, which – as is well known, and certainly in the hospitality industry – spans a large swath of occupations and trades. The staff could also include people from around the world taking a year or two to be part of a great and noble experiment while also learning a trade or serving as medical recreation/school staff. For scholars there would no longer be the constant worry of obtaining a visa in time to attend a conference, as all “citizens of the world” would be immediately admissible. For migrants and refugees, there would be a place to build a new life. Indeed, the conference centers would use the wonderful diversity of backgrounds and languages and ideas to develop cognitive and noncognitive skills, helping everyone achieve their highest potential and thereby advancing both the own good and the common good.

### Revisiting One Dimension of Personal Restriction in the United States

As a final exercise, one might venture onto perilous territory to think again about the increasing emphasis on high-skilled immigrants, a dimension of restriction based on personal characteristics. Should the United States favor the immigration of the more educated or the less educated? If the less educated are less educated due to lack of opportunities, their children will inherit their drive and energy and in the American world of opportunity will achieve much. Thoughts like this were in the minds of two members of the U.S. Select Commission on Immigration and Refugee Policy – Cabinet secretaries, heads of executive departments – when they met, as part of their regular schedule of meetings, on 18 June 1980 in The Great Hall of the Department of Justice in Washington, DC, with the Honorable Theodore M. Hesburgh, Chairman, presiding, to consider criteria for selecting new-seed independent immigrants (U.S. Select Commission on Immigration Refugee Policy, 1980b, p. 325).

– The Honorable Patricia Roberts Harris, Secretary of Health, Education, and Welfare:

“Some of the models before us suggest standards for admission in the third category – English competency, education. I have a very non-legalistic reaction to that which goes to Emma Lazarus's poem, ‘Give Me Your Huddled Masses.’ Those are not people with degrees or people who speak English. We should maintain a place in this country for people who have the ‘get up and git’ to come here.”

– The Honorable Benjamin Civiletti, Attorney General:

“I would agree with [Secretary Harris]. I'm not sure how I come up. But I do know that if we had an exclusionary system with regard to language and occupation in our historical preferences, I would not be sitting here now!”

## Author Contributions

This paper was written by GJ.

## Conflict of Interest

The author declares that the research was conducted in the absence of any commercial or financial relationships that could be construed as a potential conflict of interest.

## Footnotes

^1^These backlogs pertain to what are called “visa numbers” – visas for numerically limited permanent immigration. Of course, all applications are vulnerable to processing delays; the backlogs that arise from processing may be called visa-processing backlogs, as opposed to visa-number backlogs. The focal backlogs in this paper are visa-number backlogs.

^2^That restriction leads to unauthorized migration and visa-number backlogs and these in turn to new policy devices to address them has long been recognized and discussed, especially in historical accounts of migration restriction regimes, as will be seen in the section on The Consequences of Migration Restriction in the United States (e.g., Vialet, 1979; Masanz, 1980; U.S. Immigration and Naturalization Service, 1991; Bruno, 2001; Wasem, 2010). Social science discussions of these phenomena include Czaika and Hobolth (2016), Brekke et al. (2017), and Poston (2019).

^3^The Human Development Index dates to 1990 and the Gender Gap Index to 2006, while the Human Capital Index was introduced in 2019.

^4^Aberle et al. (1950, p. 101) define a society as “a group of human beings sharing a self-sufficient system of action which is capable of existing longer than the life-span of an individual, the group being recruited at least in part by the sexual reproduction of its members.” Thus, a tribe and a nation are societies, but a monastery is not. The elements of a self-sufficient system of action include a shared form of communication (i.e., language), shared cognitive orientations, and a shared articulated set of goals. The recruitment mechanism must yield a supply of “effectively socialized individuals from the maturing generation,” possibly supplemented by recruits acquired through “immigration and conquest,” (Aberle et al., 1950, p. 101), the latter also effectively socialized (Aberle et al., 1950, p. 109). Thus, the non-sexual recruitment practices of nation-states and the development of screening and socialization systems – as well as associated policymaking processes – constitute an important topic of study. Note that, in classic Aberle et al. (1950) terms, personal characteristics are good indicators of the probability of effective socialization into the country-specific system of action. But note also that individuals may disagree on the characteristics that render desirable a prospective immigrant, as will be illustrated below.

^5^For example, the Mission Statement for the U.S. Citizenship and Immigration Services (USCIS), an agency of the Department of Homeland Security, states: “USCIS administers the nation's lawful immigration system, safeguarding its integrity and promise by efficiently and *fairly* [italics added] adjudicating requests for immigration benefits while protecting Americans, securing the homeland, and honoring our values” (USCIS Policy Manual, Vol 1, Part A, Ch 1).

^6^Predictions 2–5 are for the special case in which the valued good is cardinal, the migrant does not pay a tax or receive a bonus at either origin or destination, and there is no economic growth from pre- to post-migration in either the origin or destination country (Jasso, 1996, p. 30–42).

^7^For example, in the aftermath of the U.S. presidential election in November 2020, a Trump supporter was quoted in the New York Times as saying, “Everything I worked for, Biden wants to give to the immigrants to help them live, when they don't do nothing but sit on their butts” (Herndon, 2020).

^8^Derivation of the just society result relies on two earlier results. The first is a theorem that states, “If an observer regards a cardinal thing as a good, then that observer implicitly regards inequality in the distribution of that thing as a bad; and if an observer regards a cardinal thing as a bad, then that observer implicitly regards inequality in the distribution of that thing as a good” (Jasso, 2017). The second is a set of results showing that the larger the number of independent-minded decisionmakers distributing a thing, the smaller the inequality in the distribution (Jasso, 2009a, 2018).

^9^The Universal Declaration of Human Rights Drafting Committee, chaired by Eleanor Roosevelt, worked in 1947-1948 to prepare the UDHR.

^10^The green card (technically, Form I-551, the Permanent Resident Card) is the paper evidence of legal permanent residence. The card is called green because it was green from 1946 to 1964; it became green again in 2010.

^11^The graph appears, for example, in U.S. Immigration and Naturalization Service (2001, p. 2) and Baugh (2020, p. 3). As for the underlying numbers (initially published in the annual reports – e.g., U.S. Commissioner General of Immigration, 1898-1932; U.S. Immigration and Naturalization Service, 1943-1978, 1979-2001; U.S. Department of Homeland Security, 2002-2019), DHS continually updates the data, as noted in U.S. Department of Homeland Security (2002-2019, p. 1), for example, revising the data for the years 1973-2004 to remove duplicates, as discussed in U.S. Department of Homeland Security (2002-2019, p. 1). Accordingly, the source data are from the most recent Statistical Yearbook, namely that for 2019; although the 2019 yearbook is not yet published, the tables are already available on the DHS website. Figure 1 includes in the total for 1976 the number admitted during the Transition Quarter 1976, when the United States changed from a July-June fiscal year to an October-September fiscal year. Of course, not everyone admitted to legal permanent residence remains in the United States; estimates of the U.S. foreign-born population are prepared by DHS and by the U.S. Census Bureau (Jasso and Rosenzweig, 2020).

^12^The *USCIS Policy Manual* can be accessed via the USCIS website (https://www.uscis.gov/policy-manual). Additional material critically useful for students of U.S. immigration may be found at the main USCIS website (http://www.uscis.gov), including a Glossary (https://www.uscis.gov/tools/glossary), the website of the Office of Immigration Statistics (OIS) at DHS (https://www.dhs.gov/immigration-statistics/), and the website of the Office of Visa Services, a unit of the U.S. Department of State (http://travel.state.gov).

^13^Staff deliberations are described in U.S. Select Commission on Immigration Refugee Policy (1980b, p. 14–15, 23–24, 281–291). For exposition of estimation procedures and each staff member's point system, see Jasso (1988b).

^14^These results suggest that having a job offer and a U.S. citizen sibling are consensually viewed as plausible indicators of effective screening and socialization into the classic Aberle et al. (1950) shared “self-sufficient system of action”.

^15^For a vivid account by a contemporary of the provisions of the Immigration Act of 1924 (also known as the Johnson-Reed Act), enacted 26 May 1924, which revised and codified the numerical restrictions first tried as an experiment in the First Quota Law of 1921 (also known as the Emergency Quota Act or the Johnson Quota Act), enacted 19 May 1921, see Parker (1924, 1925); for the 1924 Act itself, see U.S. Public Law, 68-139 (1924).

^16^See Parker (1924, p. 739, 741) for a lively account about how the two chambers of the U.S. Congress had envisioned “certificates” and “visas” and the two conceptions were in the end merged into a visa system.

^17^For example, the estimates of the foreign-born population reported in Jasso and Rosenzweig (2020) would not have been possible without the Population Estimates reports.

^18^For further information, see http://uscis.gov/history-and-genealogy/genealogy/registry-files-march-2-1929-March-31-1944.

^19^As might be expected, the single most numerous class of admission is that for spouses of U.S. citizens – e.g., 304,334 (or 29.5%) of the 1,031,765 persons granted legal permanent residence in 2019 (U.S. Department of Homeland Security, 2019). A longstanding question pertains to the nativity of the U.S. citizen sponsors of spouses. The three pieces of information currently available indicate that the estimated percentage native-born among the U.S. citizens who sponsored the immigration of their spouses declined from 80.3 in 1985, to 55.0 in 1996, and to 47.4 in 2003 (Jasso and Rosenzweig, 2006, p. 354; Jasso, 2011, p. 1300).

^20^For further information and discussion, see Vialet (1979, p. 161–351).

^21^For further detail on the legislation passed in the four Immigration Eras, see Jasso and Rosenzweig (2006).

^22^The backlog figures suggest, on average, a wait of over ten years, not counting a further period for administrative processing. Of course, the waiting times differ across visa category and origin country, as well as for new arrivals and adjustees (Jasso, 2011, p. 1307-1309).
